# Appropriate Dietary Levels of Soybean Lecithin and Krill Oil Phospholipids Promote Growth, Antioxidant Capacity, and Lipid Metabolism While Reducing Lipid Deposition in Atlantic Salmon (*Salmo salar*) Fry

**DOI:** 10.3390/ani16091393

**Published:** 2026-05-02

**Authors:** Yuting Zhang, Qingli Gong, Jinghua Chen, Ming Liu

**Affiliations:** 1 Fisheries College, Ocean University of China, Qingdao 266003, China; 2 School of Marine Science and Engineering, Qingdao Agricultural University, Qingdao 266109, China; 3 Qingdao Qicai Seed Technology Co., Ltd., Qingdao 266600, China; 4 Engineering Research Centre of Mariculture (Ministry of Education), Ocean University of China, Qingdao 266003, China

**Keywords:** feed conversion ratio, visceral lipase activity, fatty acid profile, hepatic gene expression, innate immune signaling

## Abstract

This study evaluated the effects of dietary phospholipid (PL) source and supplementation level on growth performance, antioxidant capacity, and lipid metabolism in Atlantic salmon (*Salmo salar*) fry. A 56-day feeding trial was conducted using a basal diet containing 1.76% PL and six experimental diets with an additional 1.5%, 3.0%, or 4.5% PL provided by soybean lecithin (SL) or krill oil phospholipids (KOP). Dietary supplementation with 3.0–4.5% SL and 1.5–4.5% KOP significantly improved growth performance, whereas feed conversion ratio was significantly reduced in the 3.0–4.5% SL and 3.0% KOP groups (*p* < 0.05). At equivalent inclusion levels, no significant differences were observed between SL and KOP in growth performance parameters (*p* > 0.05). PL supplementation also reduced whole-body lipid deposition and enhanced visceral lipase activity in all groups except the 1.5% SL group, while antioxidant capacity was improved in all PL-supplemented groups (*p* < 0.05). SL had no significant effect on whole-body fatty acid composition (*p* > 0.05), whereas moderate to high levels of KOP significantly altered the fatty acid profile, characterized by reduced monounsaturated fatty acids and n-6 polyunsaturated fatty acids, along with increased eicosapentaenoic acid (EPA) levels (*p* < 0.05). Transcriptomic analysis indicated that PL supplementation affected hepatic lipid metabolism, with both PL sources downregulating *apoa2-like*, while KOP induced stronger hepatic transcriptional responses related to lipid utilization and innate immune signaling than SL (*p*_adj_ < 0.05). However, gut microbiota analysis revealed no significant differences in the relative abundances of the dominant phyla or in α- and β-diversity among the control, 3.0% KOP, and 4.5% SL groups (*p* > 0.05). Overall, dietary PL supplementation promoted growth, improved antioxidant capacity, enhanced lipid metabolism, and reduced lipid deposition in Atlantic salmon fry, with KOP exerting stronger effects than SL on fatty acid composition and hepatic gene expression.

## Simple Summary

As demand for Atlantic salmon continues to grow rapidly in China, optimizing feed formulation and nutritional management for Atlantic salmon fry is becoming increasingly important for efficient and sustainable aquaculture. In this study, we examined whether two common phospholipid sources, soybean lecithin and krill oil phospholipids, could improve the growth, feed utilization, antioxidant defenses, and fat metabolism of Atlantic salmon fry. We found that appropriate levels of both ingredients in the diet promoted growth, improved feed efficiency, strengthened natural antioxidant defenses, and reduced excess fat buildup in the body and liver. Krill oil phospholipids showed stronger effects on body fatty acids, especially by increasing eicosapentaenoic acid, a beneficial omega-3 fatty acid, and induced more pronounced hepatic gene expression changes related to lipid utilization and immune function. In contrast, phospholipid supplementation had little effect on the overall gut bacterial community. Overall, both phospholipid sources supported healthier early growth in Atlantic salmon fry, while krill oil phospholipids may provide additional metabolic benefits. These findings can help improve feed formulation for salmon fry, reduce excessive lipid deposition, enhance antioxidant capacity, and provide useful guidance for more efficient and sustainable aquaculture.

## 1. Introduction

Phospholipids (PLs), a major group of complex lipids, serve as essential components of cell membrane bilayers, lipoproteins, and bile. They play critical roles in maintaining cellular structure [1], facilitating lipid transport and absorption [2], and enhancing immune function [3]. Numerous studies have shown that dietary PL supplementation is of considerable nutritional importance in fish. Supplementation with appropriate levels of PLs can significantly promote fry growth in various fish species [4,5], promote normal skeletal development [6], and influence lipid deposition in fish tissues [7,8]. In addition, PLs participate in regulating intestinal and hepatic functions. Dietary PLs can improve intestinal morphology [9], modulate gut microbial composition [10], and increase intestinal lipase activity [11,12]. Moreover, PLs effectively enhance hepatic antioxidant capacity [13] and regulate hepatic lipid metabolism, including the inhibition of lipogenesis [14,15], the promotion of fatty acid oxidation [8], and the modulation of lipid transport [16,17].

Atlantic salmon (*Salmo salar*), a high-value aquaculture species, is extensively farmed worldwide [18]. As shown by data from the Food and Agriculture Organization of the United Nations (FAO), global farmed Atlantic salmon production has increased steadily over the past decades, reaching 2.713 million tonnes in 2023, accounting for approximately 7.4% of global marine aquaculture production [19]. China’s imports of Atlantic salmon (fresh, chilled, and frozen) grew by 12.6% in 2024 and 49.6% in 2025, reaching 156,551 tonnes in 2025 [20]. However, according to the China Fishery Statistical Yearbook, China’s total production of salmonids was about 49,709 tonnes in 2024, including only 1887 tonnes of salmon [21]. This suggests that domestic aquaculture production remains insufficient to meet the rapidly increasing consumer demand. In recent years, China has gradually strengthened its development of the Atlantic salmon germplasm resources, seed production, and aquaculture industry. The 14th Five-Year Plan for National Fishery Development emphasizes the promotion of facility fisheries and aquatic breeding innovation to advance fishery modernization [22]. Currently, Atlantic salmon aquaculture in China has reached a certain scale, including land-based recirculating aquaculture systems operated by Guoxin Oriental and offshore farming represented by the “Deep Blue No.1” facility. Therefore, research on feed formulation for Atlantic salmon fry is essential for supporting the sustainable growth of Atlantic salmon aquaculture in China.

Previous research has shown that the nutritional effects of dietary PLs in Atlantic salmon are influenced by both PL sources and the developmental stage of the fish. During the fry stage of Atlantic salmon, supplementation with appropriate levels of PLs in the diet can promote growth and improve feed efficiency, alleviate lipid accumulation in intestinal and hepatic tissues, and reduce skeletal deformities [23,24,25,26]. The relatively high requirement for PLs during early life stages may be associated with the low expression of PL biosynthetic genes in developing intestinal tissue, thereby restricting lipid transport capacity [27]. As development progresses, traditional perspectives suggest that the reliance of Atlantic salmon on exogenous PLs generally declines [23,25]. However, lysophospholipid supplementation during the seawater farming stage has recently been shown to significantly improve growth performance, digestive efficiency, and hepatic metabolism [28]. These findings highlight the need to optimize PL sources and supplementation levels across production stages in Atlantic salmon.

In fish nutrition research, dietary PLs are mainly derived from terrestrial animals [29], aquatic animals [30], and plants [31]. Soybean lecithin (SL), characterized by wide availability and cost-effectiveness, has been extensively utilized in aquafeed research [32,33]. Meanwhile, krill oil phospholipids (KOP) have attracted increasing attention because of their distinct nutritional characteristics and functional potential [34,35]. Previous studies on the phospholipid requirements of Atlantic salmon fry have reported inconsistent results regarding the growth-promoting effects of KOP and SL: some studies suggest that KOP is more effective [25], whereas others found no significant difference between the two sources [26]. Given these inconsistent findings, a more systematic comparison of SL and KOP is needed to clarify their differential effects in Atlantic salmon fry. The present study investigated the influence of PL sources (SL versus KOP) and graded supplementation levels on growth, whole-body composition, and fatty acid profiles in Atlantic salmon fry. In addition, their impacts on liver lipid deposition (assessed by Oil Red O staining), digestive physiology (amylase, trypsin, lipase, and alkaline phosphatase activities), antioxidant activities (total antioxidant capacity, catalase, and superoxide dismutase), and lipid peroxidation (malondialdehyde levels) were assessed. To further understand how different PL sources affect nutrient metabolism, liver transcriptome sequencing and gut microbiota analyses were conducted in the groups showing the best growth performance. This study was designed to provide a scientific basis for optimizing dietary PL selection and supplementation strategies for Atlantic salmon fry.

## 2. Materials and Methods

### 2.1. Experimental Diets

With reference to the studies of Taylor and Jaxion-Harm et al. [25,26], seven experimental diets were prepared for Atlantic salmon fry, each containing 60% protein and 17% lipid. Dietary protein was derived mainly from brown fish meal, white fish meal, and wheat gluten, while fish oil and soybean oil supplied the lipid fraction. The ingredient composition and fatty acid composition of the experimental diets are presented in Table 1 and Table 2, respectively.

A basal diet containing 1.76% total PLs was used as the control (P0). Based on the basal formulation, total PL levels were increased by 1.5%, 3.0%, or 4.5% on a dry matter basis through supplementation with SL or KOP. Diets supplemented with SL were designated as S1, S2, and S3, whereas those supplemented with KOP were designated as K1, K2, and K3. To maintain comparable total lipid levels and fatty acid composition, SL replaced soybean oil on an equivalent lipid basis, whereas krill oil was incorporated on an equivalent lipid basis by primarily replacing fish oil and, when necessary, partially replacing soybean oil to achieve the target KOP supplementation levels. Because krill oil contains natural astaxanthin, additional astaxanthin was supplemented to ensure equal astaxanthin levels among all treatments.

All powdered ingredients were finely ground and passed through an 80-mesh sieve, and the minor ingredients were then premixed and thoroughly blended with the major ingredients in a feed mixer. Subsequently, the pre-weighed oils and the required amount of water were slowly incorporated into the mixture to form a homogeneous dough. The dough was then processed into pellets using a twin-screw extrusion puffing machine (FT36-28D, Shandong Zhennuo Intelligent Equipment Co., Ltd., Jinan, China). After cooling, the pellets were sieved sequentially through 14-, 20-, 30-, 40-, and 60-mesh screens to obtain five size grades, covering a particle-size range of < 0.25 to 1.40 mm. Once all diets had been prepared in the required pellet sizes, the pellets were dried in a ventilated oven at 80 °C, cooled to room temperature, packed in light-proof sealed bags containing desiccant, and stored in a cool place until use.

### 2.2. Experimental Fish, Husbandry, and Sampling Procedures

The feeding trial was carried out in an Atlantic salmon culture system at Qicai Seed Industry Technology Co., Ltd., Qingdao, Shandong Province, China. Before the trial, Atlantic salmon juveniles were acclimated to the rearing system for two weeks under the experimental culture conditions and diets. At the start of the experiment, the initial body weight and body length were 0.16 ± 0.01 g and 2.55 ± 0.02 cm, respectively. After a 24 h fasting period, fish were randomly distributed into seven groups, each with three replicate tanks containing 100 fish in 100 L of water. According to the recommendations of Handeland et al. [36], surface light intensity was maintained at approximately 60 lx under continuous illumination. Water was supplied through a flow-through system at an exchange rate of 1.6 L/min. During the experimental period, the average water temperature was 15.8 °C, with daily fluctuations within ±0.5 °C. Dissolved oxygen, pH, salinity, ammonia nitrogen, and nitrite were maintained at 9.5 ± 1.3 mg/L, 6.9 ± 0.2, 0.98 ± 0.10‰, 0.7 ± 0.3 mg/L, and 0.8 ± 0.2 mg/L, respectively. Atlantic salmon fry were initially fed the smallest pellet grade, and pellet size was progressively increased as they grew; feeding was carried out to apparent satiation four times daily. Feed intake was recorded daily, and uneaten feed was collected, dried, and weighed. Mortality was also monitored daily. The feeding trial lasted eight weeks.

At the end of the trial, all fish were fasted for 24 h and anesthetized with MS-222 before sampling. Fish in each tank were counted, and final body weight and body length were recorded. Fifteen fish per tank were randomly sampled for analysis of whole-body moisture, crude ash, crude protein, crude lipid, and fatty acid composition. In addition, viscera from nine fish per tank were collected for analyses of digestive enzyme activities, antioxidant-related indices, and malondialdehyde content. Liver and intestinal tissues from three fish per tank were pooled to generate one composite sample per tank for transcriptomic analysis and microbiota sequencing, respectively. All of the above samples were immediately frozen in liquid nitrogen and stored at −80 °C until further analysis. Additionally, liver tissues from three fish per tank were fixed in 10% neutral buffered formalin at room temperature in the dark for Oil Red O staining. Each treatment group contained three replicate culture tanks, and the culture tank was used as the biological replicate (*n* = 3). All experimental procedures complied with the guidelines for animal experimentation of Ocean University of China. Further details are provided in Appendix A.

### 2.3. Proximate Composition Analysis

Proximate composition of the experimental diets and whole fish was analyzed according to the Official Methods of Analysis of AOAC INTERNATIONAL, 22nd Edition (2023) [37], including moisture, crude ash, crude protein, and crude lipid. Moisture was determined by oven-drying the samples at 105 °C to constant weight. Crude protein was quantified using an automatic Kjeldahl nitrogen analyzer (Kjeltec 2200, FOSS Analytical A/S, Hillerød, Denmark). Crude lipid was measured by Soxhlet extraction. For crude ash determination, the samples were first charred on an electric heating plate and then incinerated in a muffle furnace (SX2-4-10NP, Shanghai Yiheng Scientific Instrument Co., Ltd., Shanghai, China) at 550 °C until constant weight was reached.

### 2.4. Fatty Acid Composition Analysis

Fatty acid composition was analyzed by gas chromatography (GC) following the procedure described in reference [38]. Freeze-dried diet and whole-fish samples were finely ground, and about 100 mg of each was accurately transferred into a 10 mL stoppered graduated tube. Then, 3 mL of 1 N KOH-methanol was added, and saponification was carried out in a water bath at 75 °C for 20 min. After the mixture had cooled to room temperature, 3 mL of 2 N HCl-methanol was added for methylation, followed by a further incubation at 75 °C for 20 min. Once the reaction was complete, the samples were cooled again, mixed vigorously with 1 mL of n-hexane to extract fatty acid methyl esters (FAMEs), and left overnight to allow phase separation. The samples were subsequently centrifuged at 5000 rpm for 5 min, after which the upper n-hexane phase was collected for GC analysis. A 1 μL aliquot from each sample was injected into the GC system. Separation was performed on a TRACE TR-FAME GC column (60 m × 0.25 mm × 0.25 μm; Thermo Fisher Scientific, Waltham, MA, USA). Both injector and detector were maintained at 250 °C, and the split ratio was set to 100:1. Nitrogen served as the carrier gas at a flow rate of 1.0 mL/min. Fatty acid levels were calculated by peak area normalization and expressed as percentages of total fatty acids (%TFA).

### 2.5. Analysis of Enzyme Activities and Biochemical Parameters

During the early fry stage, fish individuals are small and their organs are not fully differentiated; therefore, related studies commonly use whole-body homogenates [39,40] or visceral mass samples [41] for enzyme activity analysis. In the present study, visceral mass was therefore used as the analytical material. Samples were homogenized in phosphate-buffered saline (PBS, 0.01 M) at 4 °C with a cryogenic grinder (JXFSTPRP-24, Shanghai Jingxin Industrial Development Co., Ltd., Shanghai, China) and then centrifuged at 3000 rpm for 10 min. The resulting supernatant was collected for analysis of enzyme activities and related biochemical indices. Digestive enzymes, including amylase, trypsin, lipase, and alkaline phosphatase, as well as antioxidant enzymes (catalase and superoxide dismutase), total antioxidant capacity, and malondialdehyde levels were determined using commercial kits (Nanjing Jiancheng Bioengineering Institute, Nanjing, China) following the manufacturer’s protocols. Protein concentration for activity normalization was determined using the Bradford method. All measurements were performed using spectrophotometric methods.

### 2.6. Oil Red O Staining of Liver Sections

Liver tissues were rapidly embedded in OCT compound on a freezing stage and frozen completely. Cryosections were prepared using a cryostat (CryoStar NX50, Thermo Fisher Scientific, Waltham, MA, USA) at a thickness of 8–10 μm. After equilibration at room temperature, the sections were fixed in a general tissue fixative for 15 min, rinsed with distilled water, air-dried, stained with Oil Red O, counterstained with hematoxylin, and mounted in glycerol gelatin. All reagents used in this procedure were purchased from Servicebio (Wuhan, China). Oil Red O–stained liver sections were observed and photographed using a light microscope (Axio Scope A1 Research Grade Biomicroscope, Carl Zeiss Microscopy GmbH, Jena, Germany).

Hepatic lipid droplets were imaged and quantified using ImageJ software v1.54p (National Institutes of Health, Bethesda, MD, USA).

### 2.7. Liver Transcriptome Sequencing and Analysis

Following an integrated assessment of growth performance and feed utilization efficiency, liver samples from the control group (P0) and from the best-performing treatment groups within each PL source (*n* = 3 per group) were selected for transcriptomic analysis. Libraries were sequenced in paired-end mode (PE150, 150 bp) on an Illumina NovaSeq 6000 platform (Illumina, San Diego, CA, USA). Raw RNA-seq reads underwent quality control, and adaptor-contaminated as well as low-quality sequences were removed to generate clean, high-quality reads. The clean reads were aligned to the Atlantic salmon reference genome Ssal_v3.1 (Ensembl release 112) using HISAT2 v2.0.5. Transcript assembly and gene structure prediction were carried out with StringTie v1.3.3b. Gene functional annotation was performed based on official Ensembl annotations, supplemented with homologous annotations from Swiss-Prot and protein domain annotations from Pfam.

Gene abundance was estimated using featureCounts v1.5.0-p3. Differentially expressed genes (DEGs) were screened using DESeq2 v1.20.0 with thresholds of |log_2_FoldChange| > 1 and adjusted *p*-value (*p*_adj_) < 0.05. Gene Ontology (GO) annotation and Kyoto Encyclopedia of Genes and Genomes (KEGG) pathway enrichment analyses for the identified DEGs were carried out with the clusterProfiler package v3.8.1.

The RNA-seq dataset generated in this study has been submitted to the Genome Sequence Archive (GSA) at the National Genomics Data Center (NGDC) under accession number CRA038318 and is currently under controlled access.

### 2.8. Gut Microbiota Sequencing and Analysis

Following an integrated evaluation of growth performance and feed utilization efficiency, intestinal samples from the control group (P0) and the best-performing treatment groups within each PL source were selected for gut microbiota analysis (*n* = 3 per group). Total genomic DNA was isolated from these intestinal samples with the DNeasy^®^ PowerSoil^®^ Pro Kit (QIAGEN, Hilden, Germany). The V3–V4 region of the bacterial 16S rRNA gene was amplified with primers 338F and 806R. The resulting PCR amplicons were purified with a PCR Clean-Up Kit (Shanghai Meiji Yuhua Biomedical Technology Co., Ltd., Shanghai, China) and quantified on a Qubit 4.0 fluorometer (Thermo Fisher Scientific, Waltham, MA, USA). Sequencing libraries were prepared following standard Illumina protocols and subsequently sequenced on an Illumina NextSeq 2000 platform (Illumina, San Diego, CA, USA) in paired-end mode.

After rarefaction to the lowest sequencing depth across all samples, high-quality reads were clustered into operational taxonomic units (OTUs) at 97% sequence similarity using UPARSE (v11). Taxonomic assignment was conducted in QIIME (v1.9.1) against the SILVA rRNA database (v138). OTUs identified as chloroplast or mitochondrial sequences were discarded, and a normalized OTU abundance table was generated for downstream analyses. Rarefaction curves based on the Sobs index were used to assess whether sequencing depth was sufficient. Differences in α-diversity among groups were tested with the Kruskal–Wallis rank-sum test, followed by Dunn’s test for pairwise comparisons when appropriate. *p*-values were corrected using the false discovery rate (FDR), and statistical significance was defined as *p*_adj_ < 0.05. The same procedure was used to compare the relative abundances of dominant bacterial phyla among groups. β-diversity patterns were assessed by principal coordinates analysis (PCoA) based on Bray–Curtis distances, and group separation was further evaluated using analysis of similarities (ANOSIM). Analyses of α- and β-diversity were mainly performed in Mothur (v1.30.2) and QIIME (v1.9.1).

The gut microbiota sequencing dataset generated in this study has been submitted to the GSA at the NGDC under accession number CRA038823 and is currently under controlled access.

### 2.9. Calculations and Statistical Analysis

Growth performance parameters were calculated as follows:

Weight gain (WG, %) = 100 × (final body weight (FBW, g) − initial body weight (IBW, g))/initial body weight (IBW, g);

Specific growth rate (SGR, %/d) = 100 × [ln(FBW) − ln(IBW)]/rearing period (days);

Survival rate (SR, %) = 100 × (final number of fish)/(initial number of fish);

Condition factor (CF, g/cm^3^) = 100 × FBW (g)/[final body length (cm)]^3^;

Feed conversion ratio (FCR) = feed intake (g)/body weight gain (g).

Except for the transcriptomic and gut microbiota data, all other data were first tested for normality and homogeneity of variances using the Shapiro–Wilk test and Levene’s test, respectively. After confirming that the assumptions were met, one-way analysis of variance (ANOVA) was performed using SPSS 25.0 software, followed by Tukey’s post hoc test to evaluate differences among groups. If the assumptions of normality were not met, group differences would have been analyzed using the Kruskal–Wallis test followed by Dunn’s multiple-comparison test. All these datasets met the assumptions of normality and homogeneity of variance. Results are presented as mean ± standard error of the mean (SEM), with *n* = 3 biological replicates per group.

## 3. Results

### 3.1. Growth Performance

In the SL treatments, 1.5% supplementation (S1) did not significantly affect FBW, WG, or SGR compared with the control group (P0) (*p* > 0.05), whereas 3.0–4.5% supplementation (S2–S3) significantly increased FBW, WG, and SGR and reduced FCR (*p* < 0.05). Moreover, the S3 group exhibited significantly higher FBW, WG, and SGR than the S1 group (*p* < 0.05). In the KOP treatments, all supplementation levels (K1–K3) significantly increased FBW, WG, and SGR (*p* < 0.05), while FCR was significantly reduced only in K2 (*p* < 0.05). Neither phospholipid source nor supplementation level had a significant effect on SR or CF (*p* > 0.05). Furthermore, at equivalent supplementation levels, no significant differences were detected between the two PL sources for FBW, WG, SGR, SR, CF, or FCR (*p* > 0.05) (Table 3).

### 3.2. Proximate Composition of Whole Fish

Dietary PL supplementation exerted differential effects on the proximate composition of Atlantic salmon (Table 4). Compared with the control group (P0), whole-body crude lipid content was significantly reduced in the S2, S3, K1, K2, and K3 groups (*p* < 0.001). Compared with the P0 group, no significant differences were detected in moisture, crude ash, or crude protein contents in the phospholipid-supplemented groups (*p* > 0.05).

### 3.3. Fatty Acid Composition of Whole Fish

The whole-body fatty acid composition of Atlantic salmon fry is presented in Table 5. Compared with the control group, SL supplementation did not significantly affect the whole-body fatty acid profile of the fry (*p* > 0.05), whereas KOP supplementation produced more pronounced changes in fatty acid composition, characterized by increased SFA, n-3 LC-PUFA, and EPA contents and decreased MUFA and n-6 PUFA contents. Among SFAs, C14:0 increased significantly in all KOP groups (*p* < 0.05), C16:0 increased in the K3 group (*p* < 0.05), and C20:0 decreased in the K2 and K3 groups (*p* < 0.05). Total SFA content was significantly higher than that of the control group only in the K3 group (*p* < 0.05). Regarding MUFAs, C18:1n9c was significantly lower in the K3 group compared with the control group (*p* < 0.05), and total MUFA content was significantly reduced in the K2 and K3 groups (*p* < 0.05). The most pronounced changes were observed in PUFAs. Total n-6 PUFA content decreased in K2 and K3, with C18:2n6c also reduced in K3 (*p* < 0.05). In contrast, total n-3 PUFA and n-3 LC-PUFA contents increased in K3, while EPA was elevated in both K2 and K3 (*p* < 0.05).

### 3.4. Digestive Enzyme Activity

Compared with the control group, lipase (LPS) activity in the visceral tissues was significantly higher in the S2, S3, K1, K2, and K3 groups (*p* < 0.05). At the same supplementation level, no significant differences in LPS activity were observed between SL and KOP treatments (*p* > 0.05). No significant differences were observed among the groups in the activities of trypsin (TRY), amylase (AMS), or alkaline phosphatase (AKP) (*p* > 0.05) (Table 6).

### 3.5. Antioxidant Capacity

In the visceral tissues, all phospholipid-supplemented groups showed significantly higher total antioxidant capacity (T-AOC), catalase (CAT) activity, and superoxide dismutase (SOD) activity than the control group (*p* < 0.05), while malondialdehyde (MDA) levels were significantly lower in all phospholipid-supplemented groups except S1 (*p* < 0.05) (Table 7).

### 3.6. Hepatic Oil Red O Staining

**Figure 1:**
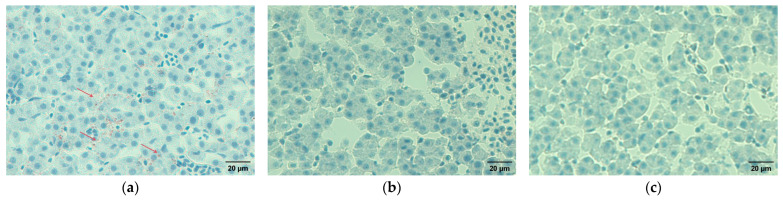
Oil Red O staining of liver sections from Atlantic salmon fed diets containing different phospholipid sources. Lipid droplets within hepatocytes are stained red (indicated by red arrows), while nuclei are counterstained blue. Scale bar = 10 μm. All groups were examined histologically. Obvious lipid deposition was observed only in the control group (P0); therefore, only P0 and the representative non-lipid deposition groups (S1 and K1) are shown. (**a**) P0 group; (**b**) S1 group; (**c**) K1 group.

Oil Red O staining revealed the presence of lipid droplets in liver sections from the control group, whereas no obvious lipid droplet accumulation was observed in the SL groups (S1, S2, and S3) or the KOP groups (K1, K2, and K3) (Figure 1).

### 3.7. Liver Transcriptome Analysis

#### 3.7.1. Overview of Transcriptome Sequencing Data

Based on growth performance and feed efficiency results, the K2 group from the KOP treatment and the S3 group from the SL treatment were selected for liver transcriptome analysis, as they represented the best-performing groups within their respective PL sources, with the highest weight gain and the lowest FCR. A total of nine RNA-seq libraries were constructed, including the P0 group (P0_1, P0_2, P0_3), the S3 group (S3_1, S3_2, S3_3), and the K2 group (K2_1, K2_2, K2_3). After adaptor trimming and removal of low-quality reads, each library generated an average of approximately 4.30 × 10^7^ clean reads, with mean Q20 and Q30 values of 98.17% and 95.26%, respectively (Table A1). Clean reads were mapped to the Atlantic salmon reference genome (Ssal_v3.1), resulting in an average mapping rate of approximately 89.90%. Overall, the sequencing data exhibited high quality and sufficient depth, supporting their suitability for subsequent differential gene expression analyses.

#### 3.7.2. Differential Gene Expression Analysis

**Figure 2:**
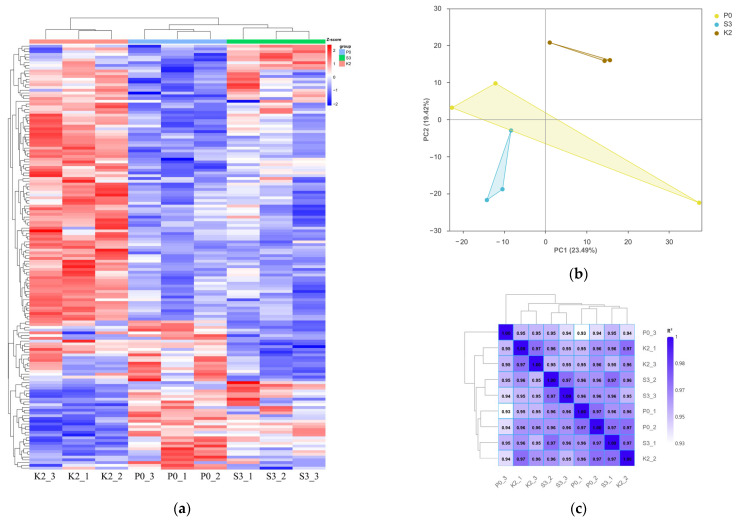
Overall expression patterns of differentially expressed genes in the liver of Atlantic salmon fed diets with different phospholipid sources. (**a**) Hierarchical clustering heatmap of differentially expressed genes; (**b**) principal component analysis (PCA) plot; (**c**) sample-to-sample correlation analysis. In the PCA plot, the shaded polygons connect biological replicates within the same treatment group and were automatically generated by the plotting software to visualize sample clustering.

Relative to P0, the S3 and K2 groups had 14 and 92 significantly upregulated genes, and 24 and 29 significantly downregulated genes, respectively (*p*_adj_ < 0.05), indicating that K2 induced a markedly stronger transcriptomic response than S3. Hierarchical clustering of the 154 DEGs identified from the three-group comparison showed clear separation by dietary treatment and high consistency among biological replicates (Figure 2a). Principal component analysis (PCA) showed that the K2 group was clearly separated from the P0 group, whereas the S3 group remained relatively closer to P0 but still exhibited a clear separation trend (Figure 2b). Notably, the treatment groups were not completely separated along the PC1 and PC2 axes, indicating that the dietary treatments induced relatively moderate rather than drastic alterations in the overall gene expression landscape. Sample correlation analysis further demonstrated strong reproducibility among biological replicates, with correlation coefficients ≥ 0.93 across all samples (Figure 2c).

#### 3.7.3. GO Enrichment Analysis

Compared with the P0 group, eight GO terms were significantly enriched in the S3 group (*p*_adj_ < 0.05), with the five most representative terms presented in Table 8 and the complete list provided in Table A2. Among these representative terms, the four most significantly enriched GO terms all involved significant downregulation of *apoa2-like* and *apoa1/a4/e-domain-like* (*p*_adj_ < 0.05). In addition, *ctgf* was significantly upregulated in the “extracellular region” term, whereas *col1a1b* and *col5a2a* were significantly upregulated in the “extracellular matrix structural constituent” term (*p*_adj_ < 0.05).

Relative to the P0 group, the K2 group displayed significant enrichment of nine GO terms (*p*_adj_ < 0.05), with the most representative terms summarized in Table 9 and the full list provided in Table A3. Among these representative terms, the “extracellular region” term showed the most pronounced enrichment, with six genes exhibiting significant expression changes: *ccl19*, *ctgf*, *mmp13*, *adm2*, and *anos1-like* were significantly upregulated, whereas *mgp-like* was significantly downregulated (*p*_adj_ < 0.05). Notably, *ccl19* was significantly upregulated in all enriched GO terms except “O-methyltransferase activity”, whereas *comtd1* and *adm2* were significantly upregulated in the “O-methyltransferase activity” and “receptor ligand activity” terms, respectively (*p*_adj_ < 0.05).

A total of one GO term was significantly enriched in the K2 group compared with the S3 group (*p*_adj_ < 0.05) (Table 10). This term was “DNA replication”, and eight genes involved in this process were all significantly downregulated in the K2 group, including *pcna-like*, *rrm1-like*, *mcm7-like*, *mcm6-like*, *orc5*, *rir1*, *pola1*, and *prim1* (*p*_adj_ < 0.05).

#### 3.7.4. KEGG Pathway Analysis

No significant enrichment of KEGG pathways was detected in the S3 group compared with the P0 group (*p*_adj_ > 0.05). However, DEGs in the K2 group relative to the P0 group were significantly enriched in the Toll-like receptor signaling pathway and the cell adhesion molecules pathway (*p*_adj_ < 0.05; Table 11). Within the Toll-like receptor signaling pathway, *tnr5*, *map3k8*, *jak1-like*, *rela*, and *hsp90ab1-like* were significantly upregulated (*p*_adj_ < 0.05). In the cell adhesion molecules pathway, *alcama-like*, *nrcam-like*, *vcam1-like*, *jam2a*, *sdc4-like*, and *tnr5* were significantly upregulated (*p*_adj_ < 0.05), whereas *esam-like* was significantly downregulated (*p*_adj_ < 0.05).

Compared with the S3 group, seven KEGG pathways were significantly enriched in the K2 group (*p*_adj_ < 0.05; Table 12), mainly involving amino acid metabolism, DNA replication, immune signal transduction, pyrimidine metabolism, phosphonate and phosphinate metabolism, and steroid biosynthesis. Among these pathways, genes involved in the RIG-I-like receptor signaling pathway were predominantly upregulated (*p*_adj_ < 0.05), whereas those involved in DNA replication and steroid biosynthesis were predominantly downregulated (*p*_adj_ < 0.05).

#### 3.7.5. Analysis of Hepatic Lipid Metabolism–Related Genes

As shown in Table 13, the lipid transport-related gene *apoa2-like* was significantly downregulated in both the S3 and K2 groups (*p*_adj_ < 0.05). In the S3 group, *apoa1/a4/e-domain-like* was also significantly downregulated, whereas the phospholipid metabolism-related gene *chdh* was significantly upregulated (*p*_adj_ < 0.05). In the K2 group, genes related to lipid droplet mobilization and remodeling, including *pnpla3* and *lpin1a*; positive regulators of lipid synthesis, including *medag-like* and *mid1ip1b-like*; and phospholipid metabolism-related genes, including *aspg*, *sgms2b*, and *pi4k2b*, were all significantly upregulated (*p*_adj_ < 0.05), whereas *msmo1-like*, which is involved in cholesterol biosynthesis, was significantly downregulated (*p*_adj_ < 0.05).

Direct comparison between the K2 and S3 groups revealed marked differences in the hepatic expression of lipid metabolism-related genes, as summarized in Table 14, with full data provided in Table A4. Compared with the S3 group, multiple genes were significantly upregulated in the K2 group (*p*_adj_ < 0.05), including genes involved in fatty acid uptake and activation (*slc27a4*, *slc27a6*, and *acsl4-like*); genes associated with fatty acid synthesis and storage (*fasn*, *acod*, *dgat1a*, and *plin2-like*), as well as positive regulators of lipid deposition (*medag-like* and *mid1ip1b-like*); genes related to phospholipid synthesis (*pcyt1b-like*, *pcyt2*, and *pisd-like*), sphingomyelin synthesis (*sgms1b* and *sgms2b*), and phospholipid remodeling and degradation (*plpp1-like* and *tmem86b*). Among genes involved in lipid transport and lipoprotein metabolism, *apoeb-like* and *lipg-like* were upregulated (*p*_adj_ < 0.05), whereas *angptl3* was downregulated in the K2 group (*p*_adj_ < 0.05). However, genes related to phospholipid/lipid transfer signaling, namely *cerk* and *c2cd2l*, were downregulated in the K2 group (*p*_adj_ < 0.05). Notably, cholesterol biosynthesis-related genes (*sqle*, *lss*, *dhcr7*, and *ebp*) were all significantly downregulated (*p*_adj_ < 0.05), whereas *cyp7a1*, a key gene involved in the conversion of cholesterol to bile acids, was significantly upregulated (*p*_adj_ < 0.05).

### 3.8. Gut Microbiota Analysis

A total of nine sets of 16S rRNA gene sequencing data were obtained in this study, including the P0 group (P0_1, P0_2, and P0_3), the S3 group (S3_1, S3_2, and S3_3), and the K2 group (K2_1, K2_2, and K2_3). All samples were rarefied to the minimum sequencing depth, retaining 40,679 high-quality sequences per sample. High-quality sequences were clustered into OTUs at 97% sequence similarity, yielding a total of 1654 OTUs after removal of chloroplast- and mitochondria-derived sequences. Rarefaction curve analysis showed that both the Sobs and Shannon indices reached a plateau at a sequencing depth of 40,679 reads, indicating that the sequencing depth was sufficient to capture the richness and diversity of the intestinal microbiota in Atlantic salmon.

**Figure 3:**
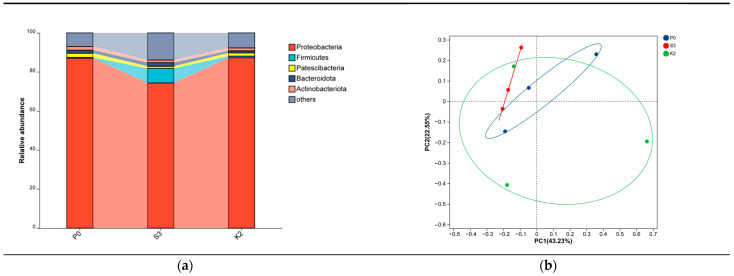
Intestinal microbiota composition and β-diversity of Atlantic salmon under different dietary phospholipid sources. (**a**) Relative abundance of intestinal microbiota at the phylum level. (**b**) Principal coordinates analysis (PCoA) based on Bray–Curtis distances (OTU level). Each point represents one sample. ANOSIM showed no significant differences among groups (R = −0.021, *p* = 0.547).

The FDR-corrected Kruskal–Wallis rank-sum test revealed no significant differences in the Sobs, Ace, Chao, Shannon, or Simpson indices among the different rearing groups (*p*_adj_ > 0.05) (Table 15). At the phylum level, the overall microbial community composition was similar among the three groups, with *Proteobacteria*, *Firmicutes*, and *Actinobacteriota* identified as the dominant phyla (Figure 3a). No statistically significant differences in the relative abundance of the major phyla were observed among groups (*p*_adj_ > 0.05). Principal coordinates analysis (PCoA) based on Bray–Curtis distances demonstrated a high degree of overlap in intestinal microbial community structures among the treatment groups, and ANOSIM analysis further confirmed that the differences among groups were not significant (R = −0.021, *p* > 0.05) (Figure 3b).

## 4. Discussion

Previous reports have shown that PLs may support the growth of fish fry. In juveniles of largemouth bass (*Micropterus salmoides*), gilthead seabream (*Sparus aurata*), and rainbow trout (*Oncorhynchus mykiss*), dietary PLs provided at appropriate levels significantly enhanced growth [9,42,43], indicating that PLs are essential nutrients in the early developmental stages of fish. This experiment also demonstrated that dietary supplementation with 3.0–4.5% SL or 1.5–4.5% KOP significantly improved the growth performance of Atlantic salmon fry, and the two PL sources showed comparable growth-promoting effects at equivalent supplementation levels. Notably, although all KOP supplementation levels improved growth performance, only the 3.0% KOP group significantly reduced FCR, suggesting that the effect of KOP on feed utilization may not continuously increase with supplementation level and that 3.0% KOP may be more favorable under the present experimental conditions. Jaxion-Harm fed Atlantic salmon fry at first feeding diets containing SL or KOP with comparable total PL contents (approximately 3.5–3.6%) and similarly observed no significant differences between the two PL sources in promoting fry growth [26]. However, Taylor reported that in larger fry (0.75–2.5 g), diets containing krill oil with 2.6% or 3.2% total glycerophospholipids resulted in significantly greater growth-promoting effects than SL diets at equivalent glycerophospholipid levels [25]. These different findings may be related to multiple factors, including fish size, basal diet composition, phospholipid molecular structure, and rearing environment.

To explore the mechanism underlying the growth-promoting effects of PL supplementation, visceral digestive enzyme activities were further measured in the fry, and both PL sources significantly increased lipase activity; similar increases have also been reported in juvenile Chinese sturgeon (*Acipenser sinensis*) [44], rainbow trout fry [5], and largemouth bass [10]. Elevated lipase activity may reflect a potential enhancement of lipid digestion by PLs, possibly increasing the availability of fatty acids for metabolic utilization. In addition to stimulating lipase activity, PLs may synergistically improve lipid digestion efficiency through multiple mechanisms, including enhancing lipid emulsification, maintaining intestinal epithelial cell membrane stability, and participating in the regulation of digestion-related neural signaling via their metabolic products [32,45,46,47]. Given that the digestive system remains immature during early fish development [48,49], dietary PL supplementation may improve the utilization efficiency of dietary lipids and thus provide more energy for fry growth, which could partially underlie the feed-efficiency- and growth-enhancing effects of exogenous PLs.

However, improved lipid digestion did not result in increased lipid deposition. Lipid content is an important indicator reflecting fat accumulation, energy reserves, and overall metabolic status in fish. In this experiment, the inclusion of 3.0–4.5% SL or 1.5–4.5% KOP significantly reduced whole-body crude lipid content and alleviated hepatic lipid deposition in Atlantic salmon fry. Similar lipid-lowering effects of PL supplementation, including reduced tissue lipid deposition [25,44,50] and lower whole-body crude lipid levels [14,44], have been reported previously. These results suggest that the absorbed lipids were not excessively stored, but were more likely transported and utilized for energy metabolism, thereby exerting a lipid-lowering effect.

This interpretation was further supported by the transcriptional changes observed in the present study. Several differentially expressed genes were associated with lipid transport, suggesting that dietary PL supplementation may alter hepatic lipid export and lipoprotein-mediated lipid trafficking. The expression of *apoa2*, which encodes a liver-expressed apolipoprotein associated with high-density lipoprotein (HDL) [51], was downregulated in the PL-supplemented groups, possibly reflecting changes in HDL assembly or metabolic demand. In addition, the significant downregulation of *apoa1/a4/e-domain-like* in the 4.5% SL group may indicate suppression of ApoA1/A4/E-related lipoprotein functions, thereby affecting HDL formation [52], chylomicron assembly [53], and receptor-mediated lipoprotein clearance [54]. Carmona-Antoñanzas et al. found that the phospholipid synthesis capacity of Atlantic salmon is limited during early developmental stages and proposed that dietary phosphatidylcholine (PC) supplementation may promote chylomicron (CM) formation [27]. This interpretation is further supported by other studies showing that dietary PLs promote the formation of lipoproteins, including CM and very low-density lipoproteins (VLDL), thereby enhancing lipid export and reducing lipid deposition in tissues [7,55]. In the present study, the downregulation of *apoa2* and *apoa1/a4/e-domain-like* may only reflect potential alterations in lipoprotein assembly and lipid transport dynamics, rather than directly indicating enhanced lipid transport. Reduced lipid accumulation may have decreased the need for compensatory apolipoprotein-mediated lipid transport, suggesting that PL supplementation may be associated with a more balanced state of lipid utilization, turnover, and storage. Although transcriptomic analysis did not reveal substantial changes in other key lipid transport genes, proteomic and lipidomic analyses may further clarify the roles of apolipoproteins in phospholipid-mediated lipid metabolism.

Studies have shown that *pnpla3* preferentially hydrolyzes PUFA-rich triglycerides, mobilizes PUFAs for phospholipid remodeling, and promotes the hepatic release of large VLDL particles, thereby maintaining the balance between hepatic lipid storage and export [56]. Lpin1 not only possesses phosphatidic acid phosphatase activity and plays a key role in triglyceride and phospholipid synthesis [57], but also facilitates hepatic fatty acid oxidation and energy expenditure by coactivating PGC-1α and PPARA [58]. Based on these findings, the significant upregulation of *pnpla3* and *lpin1* in the 3.0% KOP group may indicate that KOP enhances hepatic lipid turnover and export capacity by promoting lipid droplet remodeling, lipid mobilization, and fatty acid oxidation. In addition, the upregulation of *medag* and *mid1ip1* may reflect a hepatic response to altered lipid influx after PL supplementation, with MID1IP1 related to the positive regulation of fatty acid synthesis and MEDAG more closely associated with lipid accumulation [59,60]. Furthermore, the upregulation of *aspg*, *sgms2b*, and *pi4k2b* suggests that dietary PL supplementation may also influence phospholipid-related metabolic pathways, including lysophospholipid metabolism, sphingomyelin synthesis, and membrane lipid trafficking [61,62,63]. Taken together, these transcriptional changes suggest that dietary PL supplementation may have influenced hepatic lipid turnover, metabolic utilization, and phospholipid-related metabolic pathways, which may contribute to lipid homeostasis in Atlantic salmon fry.

In addition to modulating lipid metabolism, PL supplementation also improved the antioxidant defense and immune status of the fry, suggesting a beneficial role in maintaining physiological homeostasis. Overall, both PL sources enhanced antioxidant capacity and alleviated lipid peroxidation, as reported previously [10,64]. This beneficial effect may be attributed to the involvement of PLs in membrane architecture, which contributes to cellular protection against free radicals [65]. Moreover, by promoting fatty acid metabolism and reducing lipid accumulation, PL supplementation may alleviate oxidative stress, thereby further enhancing antioxidant capacity [50]. Beyond this general improvement in antioxidant status, KOP supplementation may also exert positive effects on innate immune processes. Transcriptome analysis showed that, compared with the P0 group, the KOP group exhibited significant enrichment of signaling pathways and functional categories related to innate immunity, particularly the Toll-like receptor signaling pathway, accompanied by significant upregulation of genes such as *tlr5*, *map3k8*, and *rela*, indicating a potential role of KOP in regulating the host innate immune response [66]. Comparisons among different PL sources further supported the potential role of KOP in innate immune regulation, with immune-related genes involved in the RIG-I-like receptor signaling pathway, including *ddx3xa*, *traf3*, and *tank*, showing higher expression levels in the KOP group. Similar immune-enhancing effects of KOP have been reported in Pacific white shrimp (*Litopenaeus vannamei*), where dietary KOP more effectively improved immune-related parameters than SL [67]. In juvenile leopard coral grouper (*Plectropomus leopardus*), krill meal supplementation was also associated with enrichment of the Toll-like receptor signaling pathway [35], further supporting the immunomodulatory potential of krill-derived ingredients. These findings collectively suggest that the immune-related transcriptional changes observed in the KOP group may be associated with their higher n-3 LC-PUFA content of KOP [68].

Furthermore, PL supplementation also altered multiple genes involved in liver extracellular microenvironment remodeling. As a typical extracellular matrix regulatory factor, *ctgf* is involved in cell proliferation, migration, adhesion, and extracellular matrix formation [69]. Its significant upregulation in both the KOP and SL groups suggests that both PL sources may participate in hepatic extracellular microenvironment and matrix remodeling. In addition, *col1a1b* and *col5a2a* were significantly upregulated in the 4.5% SL group. Since the collagens encoded by these genes are important structural components of the extracellular matrix [70], phospholipid supplementation may influence the formation of extracellular matrix structure. Meanwhile, KOP treatment also markedly influenced extracellular region-related transcripts, including *ccl19*, *mmp13*, and *mgp-like*, collectively suggesting that processes related to extracellular signaling, matrix turnover, and local homeostasis maintenance in the liver may be regulated [71,72,73]. Overall, these ECM-related transcriptional changes suggest that dietary PL supplementation, particularly KOP, may be involved in regulating hepatic extracellular matrix remodeling and local tissue homeostasis, which may have implications for liver condition.

Compared with the control group, the two PL sources exerted markedly different effects on the fatty acid profile of Atlantic salmon. Dietary KOP induced more pronounced changes, with the 3.0% and 4.5% supplementation groups showing reduced whole-body MUFA and n-6 PUFA levels, along with elevated EPA content. Additionally, the 4.5% KOP group exhibited significantly higher n-3 PUFA and n-3 LC-PUFA contents. Notably, these changes largely reflected the fatty acid profiles of the corresponding diets. In contrast, SL supplementation did not significantly alter whole-body fatty acid composition, a finding likely attributable to the similarity between the fatty acid profile of SL and that of the soybean oil it replaced. In addition, soybean lecithin-derived fatty acids may have been efficiently utilized, oxidized, or selectively incorporated into specific lipid pools, such as membrane phospholipids, rather than accumulating sufficiently to alter the overall whole-body fatty acid profile. Similar diet-reflective patterns of body fatty acid composition have also been reported in studies on pikeperch (*Sander lucioperca*), rainbow trout, and the Chinese mitten crab (*Eriocheir sinensis*) [30,74,75].

These differential whole-body fatty acid responses suggest that the two PL sources influenced lipid deposition and utilization through distinct metabolic regulatory mechanisms, which was further supported by direct comparison of the expression of liver genes involved in lipid metabolism. Compared with the 4.5% SL group, the 3.0% KOP group exhibited more pronounced transcriptional changes in hepatic lipid metabolism, suggesting alterations in fatty acid uptake and activation, lipogenesis and storage, lipoprotein assembly and transport, phospholipid synthesis and remodeling, as well as cholesterol catabolism. Specifically, the higher expression of *slc27a6*, *slc27a4*, and *acsl4-like* in the KOP group suggests more active hepatic uptake, intracellular transport, and acylation of exogenous long-chain fatty acids [76,77]. Concurrently, the significant upregulation of key lipogenic genes, including *fasn*, *dgat1a*, and *acod*, points to enhanced fatty acid synthesis, triacylglycerol assembly, and unsaturated fatty acid production [78,79,80]. This was accompanied by the upregulation of *plin2-like*, reflecting enhanced lipid droplet dynamics [81], and increased expression of positive lipogenic regulators *medag-like* and *mid1ip1b-like*, further supporting this anabolic trend. Beyond synthesis and storage, the upregulation of *apoeb-like* and *lipg-like*, coupled with the downregulation of *angptl3*, suggests that krill oil promotes hepatic lipid assembly, transport, and lipoprotein metabolism while relieving the inhibition of lipid utilization, thereby potentially contributing to altered hepatic lipid turnover [82,83,84]. Taken together, these findings suggest that the KOP group showed transcriptional features consistent with more active fatty acid synthesis, remodeling, transient storage, and subsequent export.

Notably, the apparent paradox between enhanced expression of lipid synthesis-related genes and reduced lipid deposition may be explained by the metabolic partitioning of newly synthesized fatty acids. Specifically, these fatty acids may not have been primarily stored as neutral lipids, but instead may have been preferentially directed toward phospholipid remodeling, lipid export, β-oxidation, or cholesterol-to-bile-acid conversion, thereby reflecting enhanced hepatic lipid turnover rather than simple lipid accumulation. In addition, the upregulation of genes related to LC-PUFA uptake and activation (*slc27a6*, *slc27a4*, and *acsl4-like*) and fatty acid desaturation/remodeling (*acod*) may partly explain the significantly higher whole-body EPA content in the KOP group than in the SL group, suggesting that this difference may be attributable not only to direct dietary input but also to more active hepatic fatty acid uptake, activation, remodeling, and LC-PUFA metabolism. In addition to its higher EPA and DHA contents, the stronger hepatic transcriptional response induced by KOP may also be associated with the phospholipid-bound form of EPA and DHA in krill oil, particularly within phosphatidylcholine molecular species, which may improve their bioavailability and facilitate lipid absorption, lipoprotein assembly, and membrane phospholipid remodeling [34,45,55].

Beyond these changes in hepatic fatty acid and neutral lipid metabolism, the 3.0% KOP group also displayed marked changes in pathways related to phospholipid metabolism and cholesterol homeostasis. Several genes involved in phospholipid synthesis and remodeling were significantly upregulated, including the rate-limiting enzymes for PE and PC synthesis, *pcyt2* and *pcyt1b-like* [85], the sphingomyelin synthesis-related genes *sgms1b* and *sgms2b* [86], and genes involved in phospholipid remodeling and degradation, *plpp1-like* and *tmem86b* [87,88]. This coordinated regulation suggests that KOP may influence hepatic membrane lipid renewal and phospholipid remodeling. Furthermore, the upregulation of the key bile acid synthesis gene *cyp7a1*, together with the downregulation of genes involved in de novo cholesterol synthesis (*sqle*, *lss*, *ebp*, and *dhcr7*), indicates enhanced bile acid synthesis from cholesterol and reduced endogenous sterol production in the KOP group [89,90]. Previous studies have shown that PUFAs can inhibit cholesterol biosynthesis [91]; therefore, the suppression of de novo cholesterol synthesis observed in the present study may be partly attributable to the higher level of n-3 LC-PUFA in the KOP group, particularly EPA. Moreover, the enhanced conversion of cholesterol to bile acids may facilitate improved lipid emulsification and absorption [92]. Compared with the KOP group, the 4.5% SL group exhibited specific and significant upregulation of *cerk* and *c2cd2l*, suggesting that SL may contribute more substantially to the maintenance of sphingolipid-related signaling and phospholipid transport homeostasis [93,94].

In comparison with the SL group, the KOP treatment showed enrichment of the DNA replication pathway in both GO and KEGG analyses, with key genes involved in replication origin recognition (*orc5*), DNA unwinding and replication fork progression (*mcm6-like* and *mcm7-like*), RNA primer synthesis and replication initiation (*prim1* and *pola1*), maintenance of DNA chain elongation processivity (*pcna-like*), Okazaki fragment processing (*fen1-like*), and deoxyribonucleotide supply (*rrm1-like* and *rir1*) being collectively downregulated, suggesting lower transcriptional activity related to hepatocellular DNA replication, proliferation, and renewal [95,96,97]. Meanwhile, the Pyrimidine metabolism pathway was also significantly altered, with *dck2-like* and *rir1* downregulated, whereas *cdd*, *nt5c3*, and *tymp* were upregulated, suggesting a remodeling of pyrimidine metabolism that may affect nucleoside salvage utilization and deoxyribonucleotide homeostasis, thereby influencing the supply of substrates for DNA synthesis and being consistent with the downregulation of the DNA replication pathway described above [98,99,100]. Given the comparable growth performance between the two groups, the downregulation of DNA replication-related genes more likely reflects adaptive hepatic transcriptional regulation in response to different PL sources rather than overall growth suppression. Together with the upregulation of immune-related genes and changes in lipid metabolism-related gene expression in the KOP group, this transcriptional pattern suggests that KOP may shift the hepatic functional state from one relatively oriented toward cell proliferation and renewal to one more focused on nutrient utilization, lipid metabolic regulation, and immune readiness. Overall, KOP supplementation may support normal growth while promoting metabolic and immune homeostasis through this redistribution of hepatic transcriptional activity.

In addition, the enrichment of the Cysteine and methionine metabolism and Glycine, serine and threonine metabolism pathways in the KOP group further suggests a remodeling of hepatic one-carbon metabolism and the transsulfuration pathway. In particular, the upregulation of *cbs-like* and *cth*, representing enhanced transsulfuration, suggests increased conversion of homocysteine to cysteine and a potential strengthening of glutathione/H_2_S-related antioxidant metabolism [101]. Furthermore, the upregulation of *mat2ab* together with the downregulation of *dnmt1*, *dnmt3ab*, and *mtap* suggests adjustments in methyl donor generation, DNA methylation-related processes, and the methionine salvage pathway [102,103,104]. In combination with the alterations in amino acid metabolic branches reflected by the upregulation of *sds-like* and *tdh* and the downregulation of *pipox* [105], these findings indicate that krill oil may affect hepatic amino acid metabolic networks and the way metabolic resources are allocated. Overall, the coordinated changes in one-carbon metabolism, transsulfuration, and related amino acid degradation/salvage pathways in the KOP group suggest that krill oil may contribute to supporting growth, lipid metabolic regulation, and hepatic antioxidant metabolic adaptation by modulating methyl donor utilization, redox homeostasis, and amino acid metabolic flux.

Earlier studies suggest that dietary PL supplementation may influence intestinal microbiota in fish. For instance, in largemouth bass, PL supplementation decreased the relative abundance of opportunistic pathogens, including *Klebsiella* and *Aeromonas* [10,32], whereas in rice field eel (*Monopterus albus*), it enhanced intestinal microbiota diversity and richness [106]. In contrast, the present study found that dietary PLs from different sources appeared to have limited effects on the gut microbial community structure of Atlantic salmon fry. Such inconsistencies among studies may be attributable to differences in host species, developmental stage, and rearing environment, in addition to dietary composition [107,108]. The absence of significant changes in the present study may be related to the relatively short 56-day feeding period, the potential colonization resistance of the gut microbial community during the early developmental stage of Atlantic salmon fry [109], and the balanced nutritional composition of the basal diet, which together may have weakened the modulatory effects of dietary PLs on the gut microbial community.

## 5. Conclusions

Dietary supplementation with 3.0–4.5% soybean lecithin or 1.5–4.5% krill oil phospholipids significantly improved the growth performance of Atlantic salmon fry, with 3.0–4.5% soybean lecithin and 3.0% krill oil phospholipids showing more pronounced improvements in feed utilization efficiency. In addition, phospholipid supplementation decreased whole-body crude lipid content, increased lipase activity, and enhanced antioxidant status. In terms of fatty acid composition, soybean lecithin did not significantly alter the whole-body profile, whereas krill oil phospholipids, especially at 3.0–4.5%, increased EPA and shifted the lipid profile toward higher n-3 LC-PUFA and lower MUFA/n-6 PUFA levels. Transcriptomic analysis revealed that dietary phospholipids alleviated hepatic lipid deposition by promoting lipid transport, turnover, and phospholipid metabolic remodeling. Notably, krill oil phospholipids induced stronger hepatic reprogramming than soybean lecithin, mainly reflected by enhanced responses related to fatty acid uptake and activation, phospholipid remodeling, cholesterol-to-bile-acid conversion, and RIG-I-like receptor-mediated innate immune signaling, together with reduced DNA replication-related activity. In addition, no significant effects were detected on gut microbiota α-diversity, β-diversity, or dominant phylum abundance under the present experimental conditions.

From an applied perspective, both soybean lecithin and krill oil phospholipids can be considered effective phospholipid sources in diets for Atlantic salmon fry; among them, krill oil phospholipids may have potential application value in promoting EPA deposition, improving lipid metabolic regulation, and supporting immune-related functions. However, this study has some limitations, including the relatively short feeding duration and the difficulty in obtaining sufficient blood samples from small fry, which limited the measurement of serum-related indicators. Future studies should include long-term feeding evaluations and, when fish size allows, incorporate serum lipoprotein measurements and lipidomic analyses to further clarify the long-term effects and underlying mechanisms of different phospholipid sources in Atlantic salmon fry.

## Figures and Tables

**Table 1:** Formulation and nutrient composition of the experimental diets (% dry matter).

Ingredients	P0	S1	S2	S3	K1	K2	K3
Brown fishmeal	22.00	22.00	22.00	22.00	22.00	22.00	22.00
White fishmeal	40.00	40.00	40.00	40.00	40.00	40.00	40.00
Wheat gluten	12.20	12.20	12.20	12.20	12.20	12.20	12.20
Soy protein concentrate	4.00	4.00	4.00	4.00	4.00	4.00	4.00
Pregelatinized starch	4.94	4.94	4.94	4.94	4.94	4.95	4.95
Mineral premix ^1^	1.50	1.50	1.50	1.50	1.50	1.50	1.50
NaCl	0.80	0.80	0.80	0.80	0.80	0.80	0.80
Ca(H_2_PO_4_)_2_	1.00	1.00	1.00	1.00	1.00	1.00	1.00
Vitamin premix ^2^	0.55	0.55	0.55	0.55	0.55	0.55	0.55
Antifungal agent	0.10	0.10	0.10	0.10	0.10	0.10	0.10
Antioxidant	0.05	0.05	0.05	0.05	0.05	0.05	0.05
Feeding attractant	0.20	0.20	0.20	0.20	0.20	0.20	0.20
Astaxanthin	0.009	0.009	0.009	0.009	0.006	0.003	0.000
Choline chloride	0.15	0.15	0.15	0.15	0.15	0.15	0.15
Soybean lecithin ^3^	0.00	1.56	3.13	4.69	0.00	0.00	0.00
Krill oil ^4^	0.00	0.00	0.00	0.00	3.75	7.50	11.25
Soybean oil	6.00	4.44	2.88	1.31	6.00	5.00	1.25
Fish oil	6.50	6.50	6.50	6.50	2.75	0.00	0.00
Total	100	100	100	100	100	100	100
Proximate analysis (Mean values, % dry weight)							
Crude protein	60.44	60.51	60.11	60.01	59.30	60.56	60.12
Crude lipid	17.33	17.35	17.40	17.12	17.04	16.93	17.29
Ash	14.69	14.86	14.63	14.99	15.03	15.03	15.02

The experimental diets included a control diet without phospholipid supplementation (P0), SL-supplemented diets at inclusion levels of 1.5%, 3.0%, and 4.5% (S1, S2, and S3), and krill oil-supplemented diets at the same inclusion levels (K1, K2, and K3). ^1^ Mineral premix (mg/kg diet): MgSO_4_·7H_2_O, 300.0; FeSO_4_·H_2_O, 150.0; ZnSO_4_·H_2_O, 100.0; MnSO_4_·H_2_O, 50.0; CuSO_4_·5H_2_O, 10.0; KIO_3_, 0.8; CoCl_2_, 0.3; Na_2_SeO_3_, 0.4. ^2^ Vitamin premix (mg/kg diet): vitamin A, 10.0; vitamin D_3_, 20.0; vitamin E, 500.0; vitamin K_3_, 28.0; vitamin B_1_, 56.0; vitamin B_2_, 25.0; calcium pantothenate, 100.0; niacin, 180.0; pyridoxine HCl, 50.0; biotin, 1.8; vitamin B_12_, 0.1; folic acid, 5.0; vitamin C, 500.0; inositol, 500.0. ^3^ Soybean lecithin containing 96% phospholipids was obtained from Beijing Chinaholder Biotech Co., Ltd. (Beijing, China). ^4^ Krill oil containing 40% phospholipids was obtained from Aker BioMarine ASA (Lysaker, Norway).

**Table 2:** Fatty acid composition of the experimental diets and major lipid sources (% identified fatty acids).

Fatty Acids	Diet							Lipid Source			
	P0	S1	S2	S3	K1	K2	K3	Soybean Lecithin	Krill Oil	Fish Oil	Soybean Oil
C14:0	2.81	2.90	2.99	3.09	3.71	5.16	8.06	0.08	11.12	4.38	ND ^1^
C16:0	18.49	19.35	20.32	21.37	18.36	19.13	22.79	20.90	23.83	22.35	11.00
C18:0	4.35	4.38	4.42	4.46	3.87	3.32	2.70	4.42	1.32	4.43	4.02
C20:0	0.60	0.60	0.59	0.59	0.48	0.35	0.28	0.17	ND	0.74	0.34
SFAs ^2^	26.79	28.11	29.45	30.75	27.09	29.02	35.47	26.94	38.83	34.38	15.69
C16:1n7	3.87	3.98	4.11	4.25	3.58	3.70	5.30	0.10	5.55	6.02	0.00
C18:1n9c ^3^	21.09	19.96	18.83	17.63	19.60	17.62	14.41	9.72	11.28	21.10	23.70
C20:1	2.27	2.32	2.37	2.43	1.75	1.35	1.61	0.07	0.76	3.41	0.22
MUFAs ^4^	28.97	27.99	27.08	26.13	26.11	23.15	22.45	9.88	18.86	31.66	24.09
C18:2n6c	24.24	23.51	22.82	22.06	24.77	22.05	7.73	56.80	1.97	9.02	53.60
C20:4n6	0.67	0.69	0.71	0.74	0.54	0.45	0.60	ND	0.42	1.05	ND
n-6 PUFAs ^5^	25.57	24.99	24.27	23.57	26.03	23.22	8.93	56.84	3.65	11.39	53.61
C18:3n3	3.43	3.31	3.19	3.06	3.76	3.77	2.55	6.33	2.23	1.49	6.60
C20:5n3	6.81	6.96	7.12	7.31	8.87	12.08	18.06	ND	23.00	8.05	ND
C22:6n3	8.33	8.56	8.82	9.11	8.03	8.64	12.39	ND	13.05	12.40	ND
n-3 PUFAs ^6^	18.66	18.90	19.21	19.54	20.77	24.59	33.15	6.33	38.66	22.56	6.60
n-3 LC-PUFAs ^7^	15.22	15.59	16.02	16.48	17.01	20.82	30.60	ND	36.43	21.07	ND

Dietary groups are described in Table 1. ^1^ ND, not detected; ^2^ SFAs, saturated fatty acids; ^3^ cis-fatty acids; ^4^ MUFAs, monounsaturated fatty acids; ^5^ n-6 PUFAs, n-6 polyunsaturated fatty acids; ^6^ n-3 PUFAs, n-3 polyunsaturated fatty acids; ^7^ n-3 LC-PUFAs, n-3 long-chain polyunsaturated fatty acids.

**Table 3:** Effects of different phospholipid sources and supplementation levels on growth performance in Atlantic Salmon.

	P0	S1	S2	S3	K1	K2	K3
IBW (g)	0.16 ± 0.01	0.16 ± 0.01	0.16 ± 0.01	0.16 ± 0.01	0.16 ± 0.01	0.16 ± 0.01	0.16 ± 0.01
FBW (g)	1.75 ± 0.01 ^a^	1.87 ± 0.02 ^ab^	1.98 ± 0.03 ^bc^	2.13 ± 0.04 ^c^	2.03 ± 0.06 ^bc^	2.14 ± 0.01 ^c^	2.07 ± 0.07 ^c^
WG (%)	1022.97 ± 8.53 ^a^	1099.94 ± 13.37 ^ab^	1170.32 ± 16.30 ^bc^	1271.98 ± 23.56 ^c^	1207.14 ± 39.14 ^bc^	1275.27 ± 6.18 ^c^	1233.21 ± 45.73 ^c^
SGR (%/d)	4.32 ± 0.01 ^a^	4.44 ± 0.02 ^ab^	4.54 ± 0.02 ^bc^	4.68 ± 0.03 ^c^	4.59 ± 0.05 ^bc^	4.68 ± 0.01 ^c^	4.62 ± 0.06 ^c^
SR (%)	95.85 ± 1.06	96.91 ± 1.17	97.58 ± 0.35	98.28 ± 1.24	95.51 ± 0.93	97.23 ± 0.70	97.24 ± 0.70
CF (g/cm^3^)	1.33 ± 0.15	1.28 ± 0.02	1.40 ± 0.03	1.41 ± 0.09	1.35 ± 0.02	1.34 ± 0.07	1.27 ± 0.03
FCR	0.94 ± 0.01 ^b^	0.88 ± 0.01 ^ab^	0.83 ± 0.01 ^a^	0.81 ± 0.03 ^a^	0.86 ± 0.03 ^ab^	0.82 ± 0.01 ^a^	0.85 ± 0.04 ^ab^

Dietary groups are described in Table 1. Data are presented as means ± SEM (*n* = 3). Different superscript letters within the same row indicate significant differences among groups (*p* < 0.05) based on one-way ANOVA followed by Tukey’s test.

**Table 4:** Effects of different phospholipid sources and supplementation levels on the proximate composition of Atlantic salmon (%).

	P0	S1	S2	S3	K1	K2	K3
Moisture	76.80 ± 0.15 ^ab^	76.24 ± 0.36 ^ab^	76.01 ± 0.21 ^ab^	75.78 ± 0.15 ^a^	77.03 ± 0.12 ^b^	76.28 ± 0.35 ^ab^	75.77 ± 0.15 ^a^
Ash	9.46 ± 0.47	10.32 ± 0.21	10.87 ± 0.3	10.53 ± 0.82	10.41 ± 0.26	10.88 ± 0.2	10.91 ± 0.22
Crude lipid	33.05 ± 0.62 ^c^	31.29 ± 0.22 ^bc^	29.15 ± 0.41 ^ab^	28.96 ± 0.41 ^a^	29.16 ± 0.69 ^ab^	28.39 ± 0.21 ^a^	27.31 ± 0.31 ^a^
Crude protein	57.99 ± 0.68	58.37 ± 0.61	60.72 ± 0.96	59.91 ± 0.96	59.79 ± 1.83	60.4 ± 1.41	61.74 ± 0.94

Dietary groups are described in Table 1. Data are presented as means ± SEM (*n* = 3). Except for moisture, all other proximate components are expressed on a dry matter basis. Different superscript letters within the same row indicate significant differences among groups (*p* < 0.05).

**Table 5:** Effects of different phospholipid sources and supplementation levels on the fatty acid composition of Atlantic salmon whole body (% identified fatty acids).

	P0	S1	S2	S3	K1	K2	K3
C14:0	2.35 ± 0.17 ^a^	2.67 ± 0.08 ^a^	2.48 ± 0.14 ^a^	2.72 ± 0.20 ^a^	3.50 ± 0.16 ^b^	4.52 ± 0.12 ^c^	6.71 ± 0.17 ^d^
C16:0	17.63 ± 0.87 ^a^	20.80 ± 0.92 ^ab^	19.31 ± 0.45 ^ab^	21.05 ± 1.18 ^ab^	20.19 ± 0.67 ^ab^	19.71 ± 1.05 ^ab^	23.17 ± 0.79 ^b^
C18:0	5.99 ± 0.51	6.63 ± 0.95	5.87 ± 0.26	5.78 ± 0.42	5.56 ± 0.89	4.73 ± 0.47	4.31 ± 0.53
C20:0	0.36 ± 0.00 ^bc^	0.40 ± 0.00 ^c^	0.30 ± 0.04 ^b^	0.34 ± 0.01 ^bc^	0.30 ± 0.00 ^b^	0.17 ± 0.01 ^a^	0.11 ± 0.00 ^a^
SFAs ^1^	27.59 ± 0.53 ^a^	31.41 ± 0.57 ^a^	29.50 ± 0.08 ^a^	31.03 ± 1.52 ^a^	31.06 ± 0.89 ^a^	30.54 ± 0.61 ^a^	36.14 ± 0.70 ^b^
C16:1n7	3.75 ± 0.32	4.52 ± 0.73	4.08 ± 0.59	5.11 ± 0.07	3.98 ± 0.48	3.86 ± 0.65	5.25 ± 0.36
C18:1n9c ^2^	23.54 ± 0.85 ^b^	24.50 ± 0.95 ^b^	21.84 ± 0.58 ^ab^	20.85 ± 1.05 ^ab^	23.30 ± 0.68 ^b^	20.68 ± 0.80 ^ab^	18.45 ± 0.53 ^a^
C20:1	2.14 ± 0.18	2.38 ± 0.22	2.25 ± 0.12	2.34 ± 0.28	2.03 ± 0.16	1.72 ± 0.24	1.91 ± 0.20
MUFAs ^3^	31.07 ± 0.28 ^c^	33.35 ± 1.22 ^c^	30.47 ± 0.57 ^abc^	30.15 ± 0.77 ^abc^	30.85 ± 0.17 ^bc^	27.73 ± 0.71 ^ab^	27.08 ± 0.45 ^a^
C18:2n6c	22.62 ± 0.85 ^b^	19.70 ± 0.60 ^b^	20.92 ± 0.95 ^b^	19.37 ± 0.70 ^b^	21.18 ± 1.10 ^b^	20.37 ± 0.50 ^b^	9.61 ± 0.40 ^a^
C20:4n6	0.76 ± 0.08	0.69 ± 0.07	0.82 ± 0.10	0.81 ± 0.12	0.56 ± 0.05	0.56 ± 0.09	0.61 ± 0.08
n-6 PUFAs ^4^	25.73 ± 0.31 ^c^	22.79 ± 0.61 ^bc^	22.91 ± 0.41 ^bc^	22.4 ± 1.16 ^bc^	23.47 ± 0.54 ^bc^	22.44 ± 0.47 ^b^	11.07 ± 0.66 ^a^
C18:3n3	2.39 ± 0.17	1.56 ± 0.19	2.04 ± 0.05	2.15 ± 0.22	1.74 ± 0.32	2.21 ± 0.16	2.27 ± 0.22
C20:5n3	2.49 ± 0.18 ^ab^	2.18 ± 0.20 ^a^	3.02 ± 0.17 ^ab^	2.88 ± 0.14 ^ab^	3.39 ± 0.19 ^b^	5.28 ± 0.29 ^c^	8.21 ± 0.33 ^d^
C22:6n3	10.74 ± 0.85 ^ab^	8.55 ± 0.95 ^a^	11.78 ± 0.68 ^ab^	11.24 ± 1.25 ^ab^	9.36 ± 0.80 ^a^	11.59 ± 1.15 ^ab^	15.13 ± 0.73 ^b^
n-3 PUFAs ^5^	15.62 ± 1.00 ^ab^	12.45 ± 1.16 ^a^	17.11 ± 0.93 ^ab^	16.42 ± 1.53 ^ab^	14.63 ± 1.37 ^ab^	19.28 ± 1.37 ^b^	25.72 ± 0.98 ^c^
n-3 LC-PUFAs ^6^	13.23 ± 1.03 ^ab^	10.89 ± 1.2 ^a^	15.08 ± 0.92 ^ab^	14.27 ± 1.45 ^ab^	12.89 ± 1.11 ^ab^	17.07 ± 1.54 ^b^	23.45 ± 1.08 ^c^

Dietary groups are described in Table 1. Data are presented as means ± SEM (*n* = 3). Different superscript letters within the same row indicate significant differences among groups (*p* < 0.05). Notes are the same as in Table 2.

**Table 6:** Effects of dietary supplementation with different phospholipid sources and levels on digestive enzyme activity in the viscera of Atlantic salmon fry.

	P0	S1	S2	S3	K1	K2	K3
AMS (U/mg prot)	0.28 ± 0.01	0.30 ± 0.00	0.25 ± 0.02	0.24 ± 0.01	0.25 ± 0.02	0.22 ± 0.03	0.21 ± 0.03
TRY (×10^3^ U/mg prot)	17.29 ± 0.37	19.97 ± 0.52	17.31 ± 0.47	16.22 ± 0.77	16.37 ± 1.12	16.97 ± 1.94	15.98 ± 1.14
LPS (U/g prot)	9.05 ± 0.08 ^a^	10.05 ± 0.39 ^ab^	11.04 ± 0.39 ^bc^	11.88 ± 0.58 ^bc^	11.65 ± 0.71 ^bc^	12.20 ± 0.28 ^c^	11.84 ± 0.37 ^bc^
AKP (King units/g prot)	70.09 ± 5.32	85.93 ± 4.67	77.05 ± 4.72	72.31 ± 9.06	85.54 ± 7.05	80.76 ± 5.52	66.98 ± 4.57

Dietary groups are described in Table 1. Data are presented as means ± SEM (*n* = 3). Different superscript letters within the same row indicate significant differences among groups (*p* < 0.05).

**Table 7:** Effects of dietary supplementation with different phospholipid sources and levels on oxidative stress biomarker levels in the viscera of Atlantic salmon.

	P0	S1	S2	S3	K1	K2	K3
T-AOC (U/mgprot)	12.36 ± 0.71 ^a^	17.08 ± 1.37 ^bc^	17.17 ± 1.01 ^bc^	16.73 ± 0.59 ^bc^	17.73 ± 0.83 ^bc^	18.24 ± 0.91 ^bc^	20.10 ± 0.28 ^c^
CAT (U/mgprot)	144.76 ± 3.18 ^a^	194.36 ± 8.52 ^bc^	201.46 ± 6.52 ^bc^	203.96 ± 2.77 ^bc^	203.98 ± 15.48 ^bc^	232.2 ± 10.01 ^c^	234.91 ± 15.15 ^c^
SOD (U/mgprot)	22.84 ± 0.70 ^a^	33.04 ± 2.31 ^b^	33.28 ± 0.53 ^b^	34.35 ± 1.68 ^b^	31.28 ± 2.01 ^b^	34.5 ± 0.04 ^b^	31.83 ± 1.43 ^b^
MDA (nmol/mgprot)	11.24 ± 0.38 ^e^	10.37 ± 0.23 ^de^	9.16 ± 0.23 ^cd^	7.86 ± 0.19 ^bc^	7.59 ± 0.13 ^b^	5.98 ± 0.16 ^a^	7.25 ± 0.37 ^ab^

Dietary groups are described in Table 1. Data are presented as means ± SEM (*n* = 3). Different superscript letters within the same row indicate significant differences among groups (*p* < 0.05).

**Table 8:** Representative significantly enriched GO terms in the S3 group compared with the P0 group (*p*_adj_ < 0.05).

Category	GO ID	Description	Gene Count	*p*_adj_	Upregulated Genes	Downregulated Genes
BP ^1^	GO:0042157	Lipoprotein metabolic process	2	<0.0001	–	*apoa2-like*, *apoa1/a4/e-domain-like*
BP	GO:0006869	Lipid transport	2	<0.0001	–	*apoa2-like*, *apoa1/a4/e-domain-like*
CC ^2^	GO:0005576	Extracellular region	3	0.0001	*ctgf*	*apoa2-like*, *apoa1/a4/e-domain-like*
MF ^3^	GO:0008289	Lipid binding	2	0.0014	–	*apoa2-like*, *apoa1/a4/e-domain-like*
MF	GO:0005201	Extracellular matrix structural constituent	2	0.0054	*col1a1b*, *col5a2a*	–

The S3 group represents the diet supplemented with 4.5% SL. “–“ indicates that no up- or down-regulated differentially expressed genes were detected; *apoa1/a4/e-domain-like* was annotated based on Pfam, whereas other genes labeled with “–like” were identified as highly homologous genes via Swiss-Prot; genes without the “–like” suffix have been confirmed in the Atlantic salmon genome; genes without annotation in both cases are provided with Ensembl gene IDs. Gene symbols are italicized throughout the tables and text; terms ending with “-like” are considered part of the gene symbol and are therefore italicized together with the gene name. ^1^ BP, Biological Process; ^2^ CC, Cellular Component; ^3^ MF, Molecular Function.

**Table 9:** Representative significantly enriched GO terms in the K2 group compared with the P0 group (*p*_adj_ < 0.05).

Category	GO ID	Description	Gene Count	*p*_adj_	Upregulated Genes	Downregulated Genes
CC ^1^	GO:0005576	Extracellular region	6	0.0001	*ccl19*, *ctgf*, *mmp13*, *adm2*, *anos1-like*	*mgp-like*
MF ^2^	GO:0008171	O-methyltransferase activity	1	0.0014	*comtd1*	–
MF	GO:0008009	Chemokine activity	1	0.0061	*ccl19*	–
MF	GO:0005125	Cytokine activity	1	0.0109	*ccl19*	–
MF	GO:0048018	Receptor ligand activity	2	0.0455	*ccl19*, *adm2*	–

The K2 group was supplemented with 3.0% KOP. Gene abbreviation annotations are the same as those in Table 8. “–“ indicates that no up- or down-regulated differentially expressed genes were detected. Gene symbols are italicized throughout the tables and text; terms ending with “-like” are considered part of the gene symbol and are therefore italicized together with the gene name. ^1^ CC, Cellular Component; ^2^ MF, Molecular Function.

**Table 10:** Representative significantly enriched GO terms in the K2 group compared with the S3 group (*p*_adj_ < 0.05).

Category	GO ID	Description	Gene count	*p*_adj_	Upregulated Genes	Downregulated Genes
BP ^1^	GO:0006260	DNA replication	8	0.0058	–	*pcna-like*, *rrm1-like*, *mcm7-like*, *mcm6-like*, *orc5*, *rir1*, *pola1*, *prim1*

The K2 group was supplemented with 3.0% KOP, the S3 group represents the diet supplemented with 4.5% SL. Gene abbreviation annotations are the same as those in Table 8. “–“ indicates that no up- or down-regulated differentially expressed genes were detected. Gene symbols are italicized throughout the tables and text; terms ending with “-like” are considered part of the gene symbol and are therefore italicized together with the gene name. ^1^ BP, Biological Process.

**Table 11:** Significantly enriched KEGG pathways in the K2 group compared with the P0 group (*p*_adj_ < 0.05).

KEGG ID	Description	Gene Count	*p*_adj_	Upregulated Genes	Downregulated Genes
sasa04620	Toll-like receptor signaling pathway	6	0.0068	*tnr5*, *map3k8*, *jak1-like*, *rela*, *hsp90ab1-like*, ENSSSAG00000096942	–
sasa04514	Cell adhesion molecules	7	0.0068	*alcama-like*, *nrcam-like*, *vcam1-like*, *jam2a*, *sdc4-like*, *tnr5*	*esam-like*

The K2 group was supplemented with 3.0% KOP. Gene abbreviation annotations are the same as those in Table 8. “–“ indicates that no up- or down-regulated differentially expressed genes were detected. Gene symbols are italicized throughout the tables and text; terms ending with “-like” are considered part of the gene symbol and are therefore italicized together with the gene name.

**Table 12:** Significantly enriched KEGG pathways in the K2 group compared with the S3 group (*p*_adj_ < 0.05).

KEGG ID	Description	Gene Count	*p*_adj_	Upregulated Genes	Downregulated Genes
sasa00270	Cysteine and methionine metabolism	8	0.0055	*lacc1*, *cbs-like*, *mat2ab*, *sds-like*, *cth*	*dnmt3ab*, *dnmt1*, *mtap*
sasa03030	DNA replication	6	0.0344	–	*pcna-like*, *fen1-like*, *mcm7-like*, *mcm6-like*, *pola1*, *prim1*
sasa04622	RIG-I-like receptor signaling pathway	7	0.0408	*hsp90ab1-like*, *map3k7-like*, *ddx3xa*, *cyld*, *tank*, *traf3*, *casp8-like*	–
sasa00260	Glycine, serine and threonine metabolism	5	0.0408	*tdh*, *cbs-like*, *sds-like*, *cth*	*pipox*
sasa00240	Pyrimidine metabolism	6	0.0455	*cdd*, *nt5c3*, *tymp*	*cant1*, *dck2-like*, *rir1*
sasa00440	Phosphonate and phosphinate metabolism	3	0.0492	*pcyt2*, *pcyt1b-like*, ENSSSAG00000110707	–
sasa00100	Steroid biosynthesis	4	0.0492	–	*ebp-like*, *dhcr7-like*, *sqle-like*, and *lss*

The K2 group was supplemented with 3.0% KOP. Gene abbreviation annotations are the same as those in Table 8. “–“ indicates that no up- or down-regulated differentially expressed genes were detected. Gene symbols are italicized throughout the tables and text; terms ending with “-like” are considered part of the gene symbol and are therefore italicized together with the gene name.

**Table 13:** Differential expression of lipid metabolism–related genes in the S3 and K2 groups relative to the P0 group (*p*_adj_ < 0.05).

Gene	Gene Full Name	Ensembl Gene ID	S3 vs. P0	K2 vs. P0
*pnpla3*	patatin-like phospholipase domain-containing protein 3	ENSSSAG00000077350	1.393	2.107 **
*lpin1a*	lipin 1a	ENSSSAG00000055167	1.410	2.308 ***
*msmo1-like*	methylsterol monooxygenase 1-like	ENSSSAG00000098783	−0.557	−1.430 **
*medag-like*	mesenteric estrogen-dependent adipogenesis-associated gene-like	ENSSSAG00000104028	−0.030	1.835 ***
*mid1ip1b-like*	MID1 interacting protein 1B-like	ENSSSAG00000104099	−0.348	1.216 *
*apoa2-like*	Apolipoprotein A-II-like	ENSSSAG00000072959	−1.515 ***	−0.806 *
*apoa1/a4/e-domain-like*	Apolipoprotein A1/A4/E domain-like	ENSSSAG00000101683	−1.453 ***	−0.211
*chdh*	choline dehydrogenase	ENSSSAG00000058040	1.177 *	0.311
*aspg*	asparaginase	ENSSSAG00000008371	−0.006	1.575 *
*sgms2b*	sphingomyelin synthase 2b	ENSSSAG00000045360	0.063	1.569 *
*pi4k2b*	phosphatidylinositol 4-kinase type II beta	ENSSSAG00000098343	−0.466	1.713 **

The S3 group represented the diet supplemented with 4.5% SL, and the K2 group represented the diet supplemented with 3.0% KOP. Gene abbreviation annotations are the same as those in Table 8. Gene symbols are italicized throughout the tables and text; terms ending with “-like” are considered part of the gene symbol and are therefore italicized together with the gene name. Values in the last two columns are log_2_ (Fold Change) relative to the control group. *, *p*_adj_ < 0.05; **, *p*_adj_ < 0.01; ***, *p*_adj_ < 0.001.

**Table 14:** Functional summary of lipid metabolism–related genes differentially expressed between the K2 and S3 groups (*p*_adj_ < 0.05).

Functional Category	Representative Genes	K2 vs. S3	Putative Implication
Fatty acid uptake and activation	*slc27a4*, *slc27a6*, *acsl4-like*	1.432 to 1.786	Enhanced fatty acid uptake and activation
Fatty acid synthesis and storage	*fasn*, *acod*, *dgat1a*, *plin2-like*, *medag-like*, *mid1ip1b-like*	1.340 to 1.915	Enhanced lipogenesis and lipid storage
Phospholipid synthesis and remodeling	*pcyt2*, *pcyt1b-like*, *pisd-like*, *sgms1b*, *sgms2b*, *plpp1-like*, *tmem86b*	1.010 to 2.252	Enhanced phospholipid synthesis and remodeling
Lipid transport and lipoprotein metabolism	*apoeb-like(+)*, *angptl3(−)*, *lipg-like(+)*	−1.677 to 1.322	Altered lipoprotein transport and lipid turnover
Phospholipid/lipid transfer signaling	*cerk*, *c2cd2l*	−1.458 to −1.102	Differential regulation of lipid signaling and transfer
Cholesterol and bile acid metabolism	*cyp7a1(+)*, *sqle(−)*, *lss(-)*, *ebp(−)*, *dhcr7(−)*	−1.526 to 3.573	Altered cholesterol turnover, with enhanced bile acid synthesis

The S3 group represented the diet supplemented with 4.5% SL, and the K2 group represented the diet supplemented with 3.0% KOP. Gene abbreviation annotations are the same as those in Table 8. Gene symbols are italicized throughout the tables and text; terms ending with “-like” are considered part of the gene symbol and are therefore italicized together with the gene name. The “K2 vs. S3” column represents the log_2_ (Fold Change) values of the K2 group relative to the S3 group.

**Table 15:** α-diversity indices of intestinal microbiota in Atlantic salmon fed diets containing different phospholipid sources.

α-Diversity Index	P0	S3	K2
Sobs	391.67 ± 50.50	496.33 ± 92.40	345.33 ± 87.51
Ace	415.20 ± 43.41	525.09 ± 88.27	384.30 ± 67.33
Chao	418.20 ± 48.58	527.55 ± 86.52	380.11 ± 83.62
Simpson	0.15 ± 0.17	0.10 ± 0.03	0.18 ± 0.11
Shannon	3.38 ± 0.89	3.55 ± 0.23	2.92 ± 0.93

P0 represents the control group, S3 represents the 4.5% SL group, and K2 represents the 3.0% KOP group. Values are presented as mean ± SD (*n* = 3). Differences among groups were evaluated using the Kruskal–Wallis test followed by Dunn’s test (*p*_adj_ < 0.05).

## Data Availability

The RNA-seq dataset generated in this study has been deposited in the Genome Sequence Archive (GSA) at the National Genomics Data Center (NGDC) under accession number CRA038318. The gut microbiota sequencing dataset has been deposited in the same repository under accession number CRA038823. Both datasets are currently under controlled access due to privacy protection requirements and will be made publicly available after February 2028.

## Abbreviations

ANOSIM	analysis of similarities
CAT	catalase
CM	chylomicron
CF	condition factor
DEGs	differentially expressed genes
EPA	eicosapentaenoic acid
FBW	final body weight
FCR	feed conversion ratio
GO	Gene Ontology
HDL	high-density lipoprotein
IBW	initial body weight
KEGG	Kyoto Encyclopedia of Genes and Genomes
KOP	krill oil phospholipid
LC-PUFA	long-chain polyunsaturated fatty acids
MDA	malondialdehyde
MUFA	monounsaturated fatty acids
OTUs	operational taxonomic units
PC	phosphatidylcholine
PCoA	principal coordinates analysis
PE	phosphatidylethanolamine
PL	phospholipid
PUFAs	polyunsaturated fatty acids
SFA	saturated fatty acid
SGR	specific growth rate
SL	soybean lecithin
SOD	superoxide dismutase
SR	survival rate
T-AOC	total antioxidant capacity
VLDL	very low-density lipoprotein
WG	weight gain

## Appendix A. Additional Details on Experimental Design and Bias Control

Experimental fish allocation and sample size rationale: The culture tank was used as the experimental unit. A total of 2100 fish were allocated to seven groups, with three replicate tanks per group and 100 fish per tank, resulting in 21 tanks in total. The number of fish stocked per tank was determined based on the requirements for subsequent sampling, potential mortality during the trial, and growth performance evaluation, rather than on a formal a priori sample size calculation.

Randomization and blinding: To minimize potential confounding effects, tanks from different treatment groups were assigned to positions within the rearing system using a random number method, and the order of sampling and subsequent measurements was also randomized. Owing to the practical requirements of diet preparation and feeding management, blinding was not feasible during group allocation or trial conduct, and the personnel involved were therefore aware of the group assignments. To reduce potential bias, all groups were managed under identical apparent satiation feeding and routine husbandry procedures. Furthermore, outcome assessment and data analysis were performed using random codes, which were not decoded according to the original group assignments until the stage of result presentation.

Inclusion and exclusion criteria for experimental animals: Before the start of the trial, all experimental fish were acclimated for two weeks under the same rearing conditions and experimental diets. Fish that remained in normal condition after acclimation were included in the trial following a 24 h fasting period before the formal start of the experiment. During the experiment, any animal, experimental unit, sample, or data point affected by non-treatment-related equipment failure, abnormal water quality, or inadequate sample quality was predefined for exclusion from the analysis.

Humane endpoints and monitoring: Humane endpoints were predefined in this study to avoid severe suffering or a moribund state in the experimental fish. During the rearing period, fish were monitored at least four times daily as part of routine husbandry, and the monitoring frequency was increased during periods of water quality fluctuation, handling stress, or other high-risk conditions. Monitoring indicators included swimming behavior, feeding response, body posture and balance, respiratory status, body surface and fin damage, and responsiveness to external stimuli. Fish were considered to have reached humane endpoints if they showed persistent loss of balance without rapid recovery, sustained inability to swim or feed normally, markedly reduced or absent responsiveness to stimuli, severe respiratory distress or a prolonged moribund state, or severe and irreversible body surface injury, hemorrhage, or infection. Fish reaching humane endpoints or found dead were promptly removed and recorded; dead fish were disposed of according to the harmless disposal procedures of Qingdao Qicai Seed Technology Co., Ltd.

Adverse events: No expected or unexpected adverse events occurred during the study.

**Table A1:** Statistics of transcriptome sequencing output, quality control, and read mapping in Atlantic salmon fry.

Sample ID	Clean Reads (×10^7^)	Q20 (%)	Q30 (%)	GC Content (%)	Mapping Rate (%)
P0_1	4.50	98.24	95.47	52.28	90.28
P0_2	4.13	98.17	95.19	51.94	90.01
P0_3	4.47	98.15	95.21	51.6	86.87
S3_1	4.24	98.17	95.24	51.2	90.2
S3_2	3.93	98.1	95.1	52.39	90.54
S3_3	4.14	98.16	95.23	52.87	90.57
K2_1	4.48	98.25	95.46	51.82	90.34
K2_2	4.45	98.18	95.29	51.24	90.26
K2_3	4.35	98.12	95.16	52.71	90.01
mean ± SEM	4.30 ± 0.07	98.17 ± 0.02	95.26 ± 0.04	52.01 ± 0.20	89.90 ± 0.38

**Table A2:** Significantly enriched GO terms in the S3 group compared with the P0 group (*p*_adj_ < 0.05).

Category	GO ID	Description	Gene Count	*p*_adj_	Upregulated Genes	Downregulated Genes
BP ^1^	GO:0042157	lipoprotein metabolic process	2	< 0.0001	–	*apoa2-like*, *apoa1/a4/e-domain-like*
BP	GO:0006869	lipid transport	2	< 0.0001	–	*apoa2-like*, *apoa1/a4/e-domain-like*
BP	GO:0010876	lipid localization	2	< 0.0001	–	*apoa2-like*, *apoa1/a4/e-domain-like*
BP	GO:0033036	macromolecule localization	2	0.0009	–	*apoa2-like*, *apoa1/a4/e-domain-like*
BP	GO:0071702	organic substance transport	2	0.0009	–	*apoa2-like*, *apoa1/a4/e-domain-like*
CC ^2^	GO:0005576	extracellular region	3	0.0001	*ctgf*	*apoa2-like*, *apoa1/a4/e-domain-like*
MF ^3^	GO:0008289	lipid binding	2	0.0014	–	*apoa2-like*, *apoa1/a4/e-domain-like*
MF	GO:0005201	extracellular matrix structural constituent	2	0.0054	*col1a1b*, *col5a2a*	-

The S3 group represents the diet supplemented with 4.5% SL. Gene abbreviation annotations are the same as those in Table 8. “–“ indicates that no up- or down-regulated differentially expressed genes were detected. Gene symbols are italicized throughout the tables and text; terms ending with “-like” are considered part of the gene symbol and are therefore italicized together with the gene name. ^1^ BP, Biological Process; ^2^ CC, Cellular Component; ^3^ MF, Molecular Function.

**Table A3:** Significantly enriched GO terms in the K2 group compared with the P0 group (*p*_adj_ < 0.05).

Category	GO ID	Description	Gene Count	*p*_adj_	Upregulated Genes	Downregulated Genes
CC ^1^	GO:0005576	extracellular region	6	0.0001	*ccl19*, *ctgf*, *mmp13*, *adm2*, *anos1-like*	*mgp-like*
MF ^2^	GO:0008171	O-methyltransferase activity	1	0.0014	*comtd1*	–
MF	GO:0008009	chemokine activity	1	0.0061	*ccl19*	–
MF	GO:0042379	chemokine receptor binding	1	0.0061	*ccl19*	–
MF	GO:0001664	G protein-coupled receptor binding	1	0.0061	*ccl19*	–
MF	GO:0005125	cytokine activity	1	0.0109	*ccl19*	–
MF	GO:0005126	cytokine receptor binding	1	0.0283	*ccl19*	–
MF	GO:0030545	receptor regulator activity	2	0.0455	*ccl19*, *adm2*	–
MF	GO:0048018	receptor ligand activity	2	0.0455	*ccl19*, *adm2*	–

The K2 group was supplemented with 3.0% KOP. Gene abbreviation annotations are the same as those in Table 8. “–“ indicates that no up- or down-regulated differentially expressed genes were detected. Gene symbols are italicized throughout the tables and text; terms ending with “-like” are considered part of the gene symbol and are therefore italicized together with the gene name. ^1^ CC, Cellular Component; ^2^ MF, Molecular Function.

**Table A4:** Full list of lipid metabolism–related genes differentially expressed between the K2 and S3 groups (*p*_adj_ < 0.05).

Gene	Gene Full Name	Ensembl Gene ID	K2 vs. S3	Description of Main Function
*slc27a4*	solute carrier family 27 member 4	ENSSSAG00000041801	1.432	Long-chain fatty acid transport
*slc27a6*	solute carrier family 27 member 6	ENSSSAG00000076826	1.786	Long-chain fatty acid transport
*acsl4-like*	acyl-CoA synthetase long chain family member 4-like	ENSSSAG00000056954	1.580	Activation of long-chain fatty acids to acyl-CoA
*fasn*	fatty acid synthase	ENSSSAG00000001887	1.340	De novo fatty acid synthesis
*acod*	acyl-CoA desaturase	ENSSSAG00000054075	1.366	Associated with acyl-CoA desaturation
*dgat1a*	diacylglycerol O-acyltransferase 1a	ENSSSAG00000047100	1.420	Key enzyme in triglyceride synthesis
*plin2-like*	perilipin 2-like	ENSSSAG00000040771	1.915	Lipid droplet coat protein involved in lipid storage
*medag-like*	mesenteric estrogen-dependent adipogenesis protein-like	ENSSSAG00000104028	1.863	Regulatory factor related to adipogenesis
*mid1ip1b-like*	midline 1 interacting protein 1b-like	ENSSSAG00000104099	1.558	Related to the regulation of adipogenesis
*pcyt2*	ethanolamine-phosphate cytidylyltransferase	ENSSSAG00000000721	1.010	Key enzyme in PE ^1^ synthesis pathway
*pcyt1b-like*	choline-phosphate cytidylyltransferase B-like	ENSSSAG00000078007	2.252	Key enzyme in PC ^2^ synthesis pathway
*pisd-like*	phosphatidylserine decarboxylase-like	ENSSSAG00000077607	1.865	Conversion of PS ^3^ to PE by decarboxylation
*sgms1b*	sphingomyelin synthase 1b	ENSSSAG00000078606	1.529	Sphingomyelin synthesis
*sgms2b*	sphingomyelin synthase 2b	ENSSSAG00000045360	1.504	Sphingomyelin synthesis
*plpp1-like*	phospholipid phosphatase 1-like	ENSSSAG00000066826	1.476	Dephosphorylation of lysophospholipids
*tmem86b*	transmembrane protein 86B	ENSSSAG00000084993	1.763	Lysoplasmalogenase involved in ether phospholipid metabolism
*apoeb-like*	apolipoprotein Eb-like	ENSSSAG00000086296	1.202	Apolipoprotein involved in lipid transport
*angptl3*	angiopoietin like 3	ENSSSAG00000081037	−1.677	Regulates lipoprotein lipase activity
*lipg-like*	lipase G, endothelial type-like	ENSSSAG00000053486	1.322	Endothelial lipase involved in lipoprotein lipid hydrolysis
*cerk*	ceramide kinase	ENSSSAG00000063672	−1.102	Ceramide kinase
*c2cd2l*	C2 calcium-dependent domain containing 2-like	ENSSSAG00000053874	−1.458	Related to phospholipid transport
*cyp7a1*	cytochrome P450 family 7 subfamily A member 1	ENSSSAG00000073200	3.573	Rate-limiting step in the conversion of cholesterol to bile acids
*sqle*	squalene epoxidase	ENSSSAG00000094161	−1.004	Key enzyme in cholesterol synthesis
*lss*	lanosterol synthase	ENSSSAG00000045554	−1.526	Lanosterol synthase
*ebp*	emopamil binding protein	ENSSSAG00000111808	−1.310	Related to cholesterol biosynthesis
*dhcr7*	7-dehydrocholesterol reductase	ENSSSAG00000086305	−1.003	Late step in cholesterol synthesis

The S3 group represented the diet supplemented with 4.5% SL, and the K2 group represented the diet supplemented with 3.0% KOP. Gene abbreviation annotations are the same as those in Table 8. Gene symbols are italicized throughout the tables and text; terms ending with “-like” are considered part of the gene symbol and are therefore italicized together with the gene name. The “K2 vs. S3” column represents the log_2_ (Fold Change) values of the K2 group relative to the S3 group. ^1^ PE, phosphatidylethanolamine; ^2^ PC, phosphatidylcholine; ^3^ PS, phosphatidylserine.
